# The protective effect of glycyrrhizin on hepatic ischemia-reperfusion injury in rats and possible related signal pathway

**DOI:** 10.22038/ijbms.2020.44101.10334

**Published:** 2020-09

**Authors:** Xiaoni Kou, Jiang Zhu, Xinke Xie, Mingxia Hao, Yingren Zhao

**Affiliations:** 1 Department of Infectious Diseases, the First Affiliated Hospital of Xi’an Jiaotong University, Xi’an 710000, Shaanxi Province, China; 2 Department of Hepatopathy, Affiliated hospital of Shaanxi University of Chinese Medicine, Xianyang 712000, Shaanxi Province, China; 3 Department of Galactophore, Shaanxi Provincial Tumor Hospital, Xi’an 710061, Shaanxi Province, China

**Keywords:** Apoptosis, Glycyrrhizic acid, Inflammation, Liver, Oxidative stress, Reperfusion injury

## Abstract

**Objective(s):**

To investigate the protective effect of glycyrrhizin (GL) on hepatic ischemia-reperfusion injury (HIRI).

**Materials and Methods:**

Forty SD rats were randomly divided into sham group, HIRI group, GL 100 mg/kg group, and GL 200 mg/kg group. The pathological alterations of liver tissue in each group were observed. The levels of alanine transaminase (ALT), aspartate aminotransferase (AST), endothelin-1 (ET-l), nitric oxide (NO), tumor necrosis factor-α (TNF-α), interleukin-1β (IL-1β), interleukin-6 (IL-6), superoxide dismutase (SOD), malondialdehyde (MDA), and glutathione peroxidase (GSH-Px) were detected. Western blot was used to detect the expression levels of cytoplasmic protein caspase-3, Bax, Bcl-2, heme oxygenase-1 (HO-1), nuclear factor erythroid 2-related factor 2 (Nrf2), and nuclear protein Nrf2.

**Results:**

Compared with the HIRI group, the levels of AST, ALT, ET-1, TNF-α, IL-1β, and IL-6 in GL groups were lower, serum NO content was higher, MDA content was lower, SOD and GSH-Px activities were significantly increased, apoptosis index was lower (*P<*0.05), which was more obvious in high-dose GL (200 mg/kg) group. The LC3-II/LC3-I ratio and Beclin-1 protein expression levels in the GL group were significantly lower than the HIRI group, but the expression levels of cytoplasmic protein HO-1 and nuclear protein Nrf2 were significantly higher than those of the HIRI group, which was more obvious in the high-dose GL group (*P<*0.05).

**Conclusion:**

GL has a protective effect on the liver of HIRI rats, and its mechanism may be related to activation of the Nrf2/HO-1 signaling pathway, inhibition of oxidative stress, inflammation, autophagy, and apoptosis.

## Introduction

Hepatic ischemia-reperfusion injury (HIRI) widely exists in clinical procedures such as liver resection, organ transplantation, trauma, and shock. HIRI can cause liver function damage and liver failure, which directly affects the operative successful rate and prognosis of patients (1). Current studies suggest that HIRI often leads to cell injury in the hepatic sinus, which induces the accumulation of reactive oxygen radicals, releases pro-inflammatory cytokines, causes hepatic cell necrosis, and also causes apoptosis and autophagy of hepatic cells, leading to abnormal liver function (2, 3). Therefore, the prevention and treatment of HIRI have always been an important research field in liver surgery. Various Chinese traditional medicines have been reported that play an important role in these kinds of ischemia-reperfusion injury. Glycyrrhizin (GL), whose chemical formula is C_42_H_62_O_16_ and known as glycyrrhizic acid, is one of the active constituents extracted from the traditional Chinese medicine *Glycyrrhiza uralensis*. GL has many pharmacological effects, such as anti-inflammatory, anti-oxidative, anti-apoptotic, and immune regulation, and has been reported as an inhibitor of High-mobility group protein-1 (HMGB1) in many pathological conditions. There is evidence that GL has a protective effect on ischemia-reperfusion injury of various organs (4-6). Meanwhile, current studies have shown that GL could attenuate liver injury by inhibiting TNF-α-induced hepatocyte apoptosis and oxidative stress (7, 8) and attenuate tissue damage and Kupffer cells pyroptosis during HIRI (9). So, it could be speculated that GL may have some positive effect on HIRI, however, this opinion has not yet been verified and the mechanism is not clear. This study aimed to explore the effect of GL on HIRI and the related mechanism preliminarily. 

## Materials and Methods

***Animals***

Male Sprague-Dawley rats (8~10 weeks old, 220~250 g body weight, Experimental Animal Center of Zhengzhou University) were kept under constant temperature (20~22 ^°^C) and humidity (40~50%) with a 12 hr light/dark cycle, and fed a standard diet and tap water. 

***Drugs and reagents***

GL (Minophagen Pharmaceutical Co., Ltd.). Endothelin-1 (ET-l) kit, nitric oxide (NO) kit, malondialdehyde (MDA) kit, SOD kit, GSH-Px kit, tumor necrosis factor-α (TNF-α) kit, interleukin-1β (IL-1β) kit, and interleukin-6 (IL-6) kit were purchased from Beijing solarbio science ﹠technology Co., Ltd. Caspase-3 antibody, Bax antibody, LC3 antibody Beclin-1 antibody, HO-1 antibody, Nrf2 antibody, PCNA antibody, and β-actin were purchased from Abcam, UK.

***Groups***

40 rats were randomly divided into sham group, HIRI group, GL 100 mg/kg group, and GL 200 mg/kg group, with 10 rats in each group. All animal experiments were approved by the Ethics Committee of Shaanxi University of Chinese Medicine Affiliated Hospital.

***HIRI modeling and GL intervention***

Rats were fasted for 12 hr before the operation, and rats in GL groups were injected GL 100 mg/kg and 200 mg/kg 1 hr before modeling, while sham group and HIRI group were injected with equal volume normal saline.

After being anesthetized by 1% pentobarbital sodium (50 mg/kg) intraperitoneal injection, the rats were fixed on the 37 ^°^C thermostatic operating plate. The liver was exposed through a longitudinal abdominal midline incision. The portal vein and hepatic artery were clamped with a non-invasive vascular clamp to make the middle and left liver lobes ischemic (about 70%). After 90 min, the clamp was released to restore liver blood flow for reperfusion, and the abdomen was closed by suture. The success criteria of hepatic ischemia were gray liver surface color and soft liver texture, and the success criteria of reperfusion were restored ruddy liver surface color. On the sham group was conducted the same operations as the HIRI group except clamping hepatic arteries and veins. After 6 hr of reperfusion, all rats were executed by drawing-out heart blood and liver tissue was collected under aseptic condition and frozen at -80 ^°^C for later use.

***Pathological examination of the liver***

A part of liver tissues was fixed in 10% formalin, embedded in paraffin, sectioned, and stained with Hematoxylin and Eosin (HE). The morphology of liver tissues was observed under an ordinary optical microscope. The degree of liver injury was evaluated according to the SUZUKI grading standard(10). The SUZUKI grading standard contains 5 grades (0~4) according to tissue congestion, cell ballooning, and necrosis area. No tissue congestion, ballooning, and necrosis were recorded as grade 0, small tissue congestion, ballooning, and necrosis were recorded as grade 1, mild tissue congestion, ballooning, and necrosis were recorded as grade 2, moderate tissue congestion, ballooning, and necrosis were recorded as grade 3, severe tissue congestion, ballooning, and necrosis over 60% of the area were grade 4. The grades among all groups were compared.

***Determination of liver function and liver injury indexes***

The supernatant was obtained after centrifugation. The serum levels of alanine transaminase (ALT) and aspartate aminotransferase (AST) were detected by the automatic biochemical analyzer, and the levels of plasma ET-l and serum NO were detected using the ELISA kit.

***Determination of inflammatory cytokines***

Serum levels of inflammatory cytokines TNF-α, IL-1β, and IL-6 were detected according to the operating instructions of the ELISA kit.

***Detection of oxidative stress indicators***

The liver tissues of each group were made into homogenates. The content of MDA was determined by the thiobarbituric acid method, the activity of SOD was measured by the xanthine oxidase method, and GSH-Px activity was determined by the dithiodinitrotoluidine method, which was carried out in strict accordance with the operating instructions of the kit.

***Observation of apoptosis by TUNEL staining***

The frozen liver tissue was cut into 6 μm sections on the slicing machine. After overnight fixing with 4% paraformaldehyde, the liver tissue was stained with a TUNEL cell apoptosis detection kit, which was carried out in strict accordance with the operating instructions of the kit. Apoptosis was observed under the fluorescence microscope. Apoptotic cells were brown, normal cells were blue-purple, apoptosis index (AI) = apoptotic cells / total cells * 100%.

***Western blot***

The nuclear proteins and cytoplasmic proteins were extracted from liver tissues by nuclear proteins and cytoplasmic proteins extraction kits, respectively, strictly according to the instructions of the kits. Cytoplasmic proteins were used to detect the expression of Caspase-3, Bax, Bcl-2, LC3, Beclin-1, Nrf2, and HO-1 proteins, and nuclear proteins were used to detect the expression of Nrf2 proteins. Protein concentration was determined by the BCA method. 40 μg protein samples were separated by polyacrylamide gel electrophoresis and were transferred to polyvinylidene fluoride membranes (PVDF membrane). Blocking at room temperature with skimmed milk powder at room temperature for 2 hr, and then were incubated with Bax, Bcl-2, Caspase-3, LC3, Beclin-1, HO-1, Nrf2, PCNA and β-actin (1:1000) antibodies at 4 ^°^C overnight. Subsequently, the membranes were incubated with the corresponding secondary antibody IgG (1:2000) for 1 hr at room temperature. After chemiluminescence development, image J was used to calculate the ratio of the target band and internal reference band gray value, that is, the relative expression of the target protein.

***Statistical analysis***

All data were analyzed using SPSS 19.0 and presented as mean±standard deviation ( ±*s*). Data between multiple groups were calculated with one-way ANOVA, pairwise comparison was performed by the *LSD* test. Results between the two groups were analyzed with an independent sample *t-*test. Statistical significance was accepted for *P-*value<0.05.

## Results

***Effects of GL on pathological findings in the liver***

**Figure 1:**
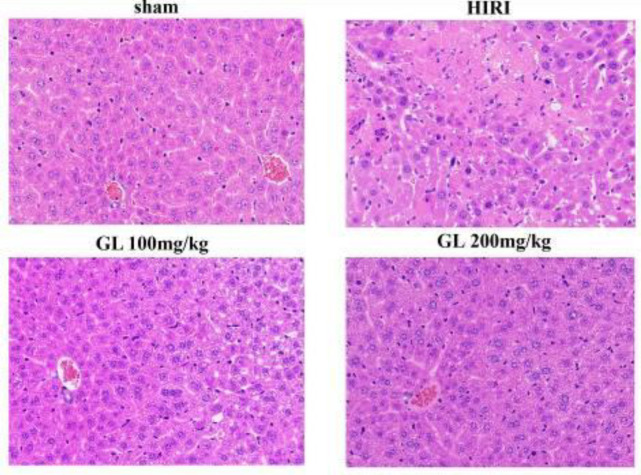
Pathological alterations of liver tissue in each group (magnification, ×200)

HIRI: Hepatic ischemia-reperfusion injury; GL: Glycyrrhizin

HE staining showed that, compared with the sham group, liver cells in the HIRI group were patchy necrosis, liver cells around the central vein were moderate and severe edema, obvious hepatic sinus block, and inflammatory cell infiltration. Compared with the HIRI group, liver cells in the GL groups showed focal or spot-like necrosis, mild edema of liver cells around the central vein, hepatic sinus block, and attenuated inflammatory cell infiltration (Figure 1). The SUZUKI score in the HIRI group was 3.60±0.49, and those in GL 100 mg/kg and 200 mg/kg groups were 2.50±0.67 and 1.90±0.70, respectively, which were both lower than that of the HIRI group (both *P*<0.05).

***Effects of GL on liver function and liver injury indexes***

Compared with the sham group, the levels of AST, ALT, and ET-1 in the HIRI group and GL group were significantly increased, while the serum level of NO was significantly reduced (*P*<0.05). Compared with the HIRI group, the levels of AST, ALT, and ET-1 in the GL group were significantly reduced, while the level of NO was significantly increased, and the changes in the GL 200 mg/kg group were more obvious (*P*<0.05, Table 1).

***Inhibition of GL on inflammation***

Compared with the sham group, the serum levels of TNF-α, IL-1β, and IL-6 in the HIRI group and GL group were significantly increased (*P*<0.05, Table 2). Compared with the HIRI group, the levels of TNF-α, IL-1β, and IL-6 in the GL group were significantly decreased, and the changes in GL 200 mg/kg group were more obvious (*P*<0.05, Table 2).

***Inhibition of GL on the oxidative stress response***

Compared with the sham group, MDA content in the HIRI group was significantly increased, while the activities of antioxidant enzymes SOD and GSH-Px were significantly decreased (*P*<0.05, Table 3). Compared with the HIRI group, MDA content in the GL group was significantly decreased, the activities of antioxidant enzymes SOD and GSH-Px were significantly increased, and the changes in GL200 mg/kg group were more obvious (*P*<0.05, Table 3).

***GL improved apoptosis of hepatocytes in HIRI rats***

**Figure 2:**
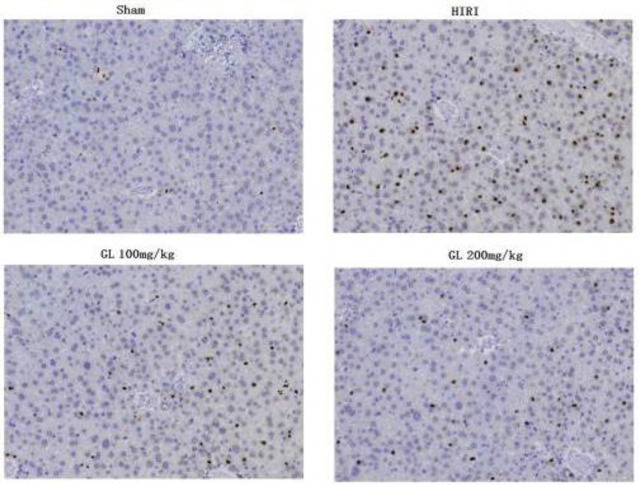
Apoptosis of hepatocytes in each group of rats (magnification, ×200)

HIRI: Hepatic ischemia-reperfusion injury; GL: Glycyrrhizin

TUNEL staining showed that only a few apoptotic positive cells were observed in the Sham group, and a large number of positive cells with yellow staining were observed in the HIRI group. The positive cells in the GL group were significantly less than those in the HIRI group (Figure 2). There were statistically significant differences in AI between each group (*P*<0.05, Table 4).

**Figure 3:**
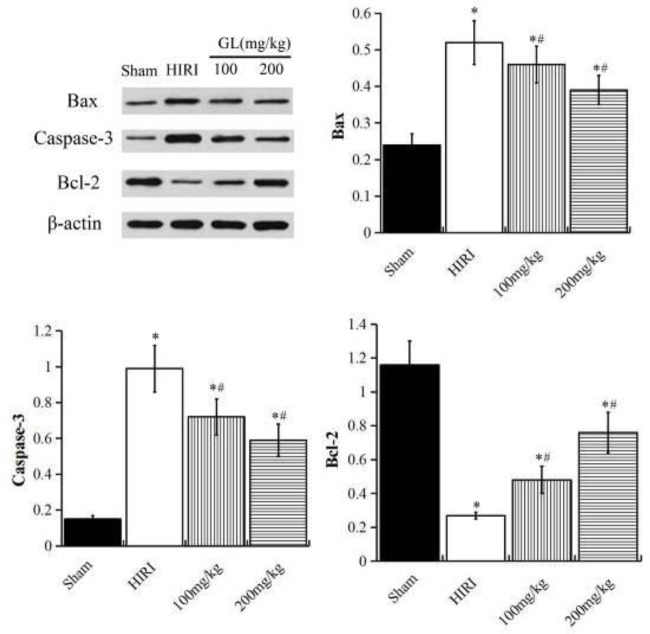
Effects of GL on the expression of apoptosis-related proteins. Compared with the Sham group, ^*^P<0.05; compared with the HIRI group, ^#^*P<*0.05

HIRI: Hepatic ischemia-reperfusion injury; GL: Glycyrrhizin

Western blot showed that the expression levels of Caspase-3 and Bax protein in the HIRI group were significantly higher than the sham group, while the expression levels of Bcl-2 protein were significantly lower than the sham group (*P*<0.05). Meanwhile, the expression levels of Caspase-3 and Bax protein in the GL group were significantly lower than the HIRI group, while the expression levels of Bcl-2 protein were significantly higher than the HIRI group, and the changes were more obvious in the GL 200 mg/kg group (*P*<0.05, Figure 3).

***Effect of GL on the expression of autophagy-related proteins LC3 and Beclin-1***

**Figure 4:**
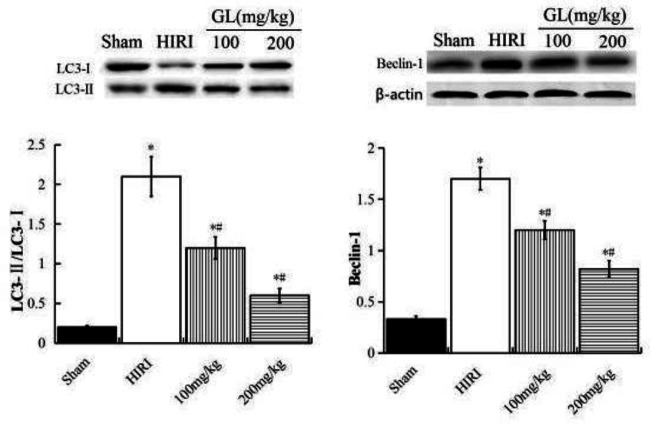
Effects of GL on the expression of autophagy-related proteins. Compared with the Sham group, ^*^P<0.05; compared with the HIRI group, ^#^*P<*0.05

HIRI: Hepatic ischemia-reperfusion injury; GL: Glycyrrhizin

Western blot showed that the LC3-II/LC3-I ratio and Beclin-1 protein expression levels in the liver tissues of the HIRI group were significantly higher than the sham group (*P*<0.05). Meanwhile, the LC3-II/LC3-I ratio and Beclin-1 protein expression levels in the liver tissue of the GL group were significantly lower than the HIRI group (*P*<0.05), and the changes were more obvious in the GL 200 mg/kg group (*P*<0.05, Figure 4)

***Effect of GL on the Nrf2/HO-1 signal pathway***

**Figure 5:**
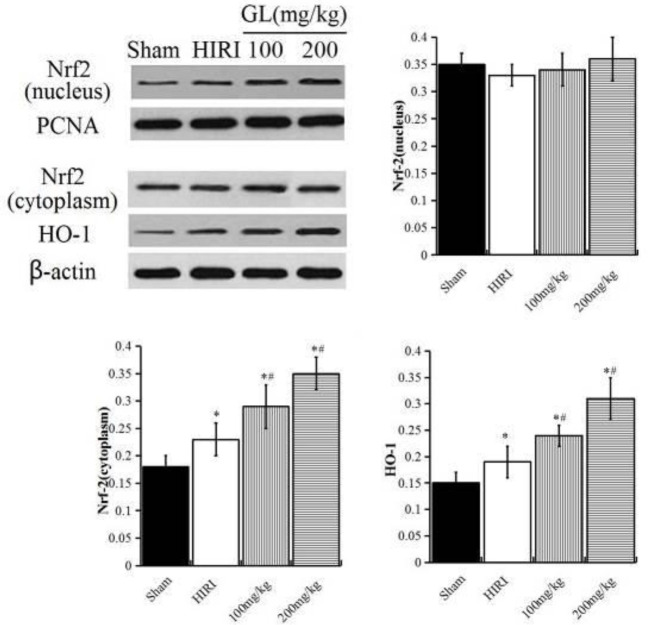
The effect of GL on the expression of Nrf2 and HO-1 protein. A: Nuclear protein, B: Cytoplasmic protein. Compared with the Sham group, ^*^P<0.05; ; compared with the HIRI group, ^#^*P<*0.05

HIRI: Hepatic ischemia-reperfusion injury; GL: Glycyrrhizin

The molecular mechanism of GL protecting the liver was explored by detecting the expression levels of Nrf2/HO-1 pathway-related proteins. There was no statistically significant difference in the expression of cytoplasmic protein Nrf2 between the sham group, HIRI group, and GL group (*P*>0.05). Compared with the sham group, the expression levels of cytoplasmic protein HO-1 and nuclear protein Nrf2 were significantly increased (*P*<0.05). Compared with the HIRI group, the expression levels of cytoplasmic protein HO-1 and nuclear protein Nrf2 were significantly increased, and the changes were more obvious in the GL200 mg/kg group (*P*<0.05, Figure 5). 

## Discussion

HIRI is an important cause of liver dysfunction after liver surgery, liver failure, and primary liver transplantation without function. Current studies have found that the mechanism of HIRI may be related to various factors, such as oxygen-free radical production, inflammatory reaction, calcium overload, and cell apoptosis (11, 12). Under normal physiological conditions, inflammatory and anti-inflammatory reactions, oxidation, and anti-oxidation systems are in a dynamic equilibrium state. After ischemia-reperfusion, neutrophils infiltrate the inflammatory injury site, which can promote the release of inflammatory cytokines and stimulate inflammatory cascade reaction (13). At the same time, a large number of reactive oxygen species (ROS) are produced in liver tissues, which react with lipids to produce a large amount of lipid peroxides (MDA) and reduce the activities of antioxidant enzymes such as SOD and GSH-Px, resulting in an imbalance of tissue oxidation and antioxidant system, eventually leading to tissue damage and necrosis. The results of this study suggested that HIRI could promote inflammatory response and apoptosis, weaken the antioxidant ability, and cause liver tissue damage and necrosis, which is consistent with the present study (14-16). Meanwhile, autophagy levels will increase in HIRI(17, 18). At present, many autophagy associated genes (ATG) involved in autophagy have been identified such as LC3 and Beclin-1, which is the earliest positive regulator of autophagy. LC3-II is a specific marker of the autophagic membrane, which is located in pre-autophagosome and autophagosome. By detecting the change of the LC3-II/LC3-I ratio, the intensity of autophagic activity can be determined(3). The results of this study showed that the LC3-II/LC3-I ratio and Beclin-1 protein were highly expressed in HIRI tissue, suggesting that the autophagy level in HIRI tissue was increased.

GL is the main physiological active substance extracted from the root of *G. uralensis*. It has many biological activities, such as anti-oxidant, anti-inflammatory, and immune enhancer. Studies have shown that GL can inhibit the release of pro-inflammatory factors, reduce MDA content, improve the activity of antioxidant enzymes, maintain the balance of free radicals/oxidants and antioxidants in the intracellular environment, inhibit apoptosis, and alleviate ischemia-reperfusion injury (4, 19, 20). Yan T *et al*. showed that GL could weaken liver injury by inhibiting hepatocyte apoptosis, which could be used against acetaminophen (APAP) overdose (8). Sil *et al. *indicated that GL treatment could improve the hepatocellular damage in metabolic syndrome rats by inhibiting oxidative stress, inflammation, and cell apoptosis (21). The same effect of GL was observed in hepatic injury induced by LPS/D-galactosamine (22). The results of this study showed that GL could significantly reduce the necrosis of liver cells, reduce hepatic sinus block, decrease inflammatory cell infiltration, reduce the inflammatory reaction and oxidative stress induced by HIRI, improve antioxidant capacity, alleviate HIRI, and protect liver tissue. Besides, GL could inhibit liver cell apoptosis, which was consistent with the existing research results. 

Autophagy is a pathway to maintain homeostasis by transporting damaged aging or denatured proteins and organelles to lysosomes for digestion and clearance (17). However, excessive autophagy can lead to excessive degradation of normal proteins and organelles and damaged cells (18). The present study showed that some natural products such as resveratrol, berberine, and curcumin can trigger autophagy via canonical (Beclin-1-dependent) and non-canonical (Beclin-1-independent) pathways. A study showed that coptisine treatment increased cell survival, inhibited apoptosis, and reduced the protein level of LC3-II, cleaved Caspase-3, Beclin1, and Sirt1, suggesting that coptisine may protect cardiomyocyte damage by H/R through suppressing autophagy (23). Meanwhile, results of a study show that pretreatment with glycyrrhizin significantly reduced 3-NP–mediated activation of autophagy marker LC3-II (24). The results of this study showed that GL could up-regulate the expression of Beclin-1 and LC3-II/LC3-I ratio in HIRI tissue, suggesting that GL may inhibit the formation and maturation of autophagosomes by inhibiting the conversion of LC3-I to LC3-II and the synthesis of Beclin-1, thus alleviating HIRI in rats.

Nrf2 is a leucine zipper transcription factor in the alkaline region. It is also a regulator of many physiological processes, such as anti-oxidant, anti-inflammatory, and anti-apoptotic (25). The signaling pathway composed of Nrf2, cytoplasmic protein Keap1, and antioxidant response sequence element (ARE) has the effect of antioxidant stress damage in many tissues and organs. In normal physiological conditions, Nrf2 binds to Keap1, which makes Nrf2 in an inhibitory state. When oxidative stress occurs, Nrf2 is activated, and the activated Nrf2 is released from Keap1 and transferred to the nucleus, which combines with ARE in the nucleus, up-regulates the expression of downstream phase II detoxifying enzymes and antioxidant genes, and enhances the activity of antioxidant enzymes, to realize the effect of antioxidant stress (26, 27). HO-1 is a small molecular heat shock protein, which can catalyze the degradation of heme to produce biliverdin, CO and Fe^2+^, which together form an endogenous protective system against oxidative stress injury, weaken oxidative stress and inflammatory response, inhibit the expression of pro-apoptotic proteins, to exert its anti-apoptotic effect (28). Activation of the Nrf2 signaling pathway can induce the up-regulation of HO-1 expression, inhibit the formation and release of inflammatory mediators, exert anti-inflammatory, antioxidant and anti-apoptotic effects, protect cells (29). The results of this study showed that GL could activate Nrf2, promote the nuclear transfer of Nrf2 protein, up-regulate the expression of HO-1, inhibit oxidative stress response, and protect liver tissue.

**Table 1:** The levels of liver injury indexes in each group of rats

Group	AST (U/L)	ALT (U/L)	NO (μmol/L)	ET-1 (ng/L)
Sham	122.20±8.28	79.87±8.86	109.11±6.71	52.11±4.79
HIRI	543.76±16.62^*^	348.19±10.74^*^	45.27±4.03^*^	158.22±7.00^*^
GL 100 mg/kg	349.74±12.24^*#^	258.04±22.23^*#^	64.34±5.47^*#^	126.99±6.52^*#^
GL 200 mg/kg	243.92±11.03^*#^	139.67±7.13^*#^	82.74±6.59^*#^	97.30±5.72^*#^

Compared with Sham group, ^*^P<0.05; compared with HIRI group, ^#^*P<*0.05 AST: Aspartate aminotransferase; ALT: Alanine transaminase ; ET-l : Endothelin-1; NO: Nitric oxide; HIRI: Hepatic ischemia-reperfusion injury; GL: Glycyrrhizin

**Table 2:** The levels of inflammatory cytokines in each group of rats

Group	TNF-α (ng/L)	IL-1β (ng/L)	IL-6 (ng/L)
Sham	42.59±4.66	63.41±4.85	34.38±2.53
HIRI	183.42±7.14^*^	234.03±10.36^*^	134.05±5.21^*^
GL 100 mg/kg	156.11±6.02^*#^	187.94±9.53^*#^	104.57±6.77^*#^
GL 200 mg/kg	113.71±5.34^*#^	141.19±6.51^*#^	64.01±2.88^*#^

Compared with Sham group, ^*^P<0.05; compared with HIRI group, ^#^*P<*0.05

TNF-α: Tumor necrosis factor-α, IL-1β: Interleukin-1β, IL-6: Interleukin-6; HIRI: Hepatic ischemia-reperfusion injury; GL: Glycyrrhizin

**Table 3:** The levels of oxidative stress indicators in each group of rats

Group	MDA (nmol/mL)	SOD (U/mL)	GSH-Px (U/mL)
Sham	5.75±0.31	122.46±7.81	185.99±7.17
HIRI	19.64±1.95^*^	56.87±4.05^*^	121.21±5.60^*^
GL 100 mg/kg	14.01±1.43^*#^	76.78±4.38^*#^	143.27±5.26^*#^
GL 200 mg/kg	9.63±0.57^*#^	104.18±5.24^*#^	170.09±6.87^*#^

Compared with Sham group, ^*^P<0.05; compared with HIRI group, ^#^*P<*0.05

SOD: Superoxide dismutase; MDA: Malondialdehyde, GSH-Px: Glutathione peroxidase; HIRI: Hepatic ischemia-reperfusion injury; GL: Glycyrrhizin

**Table 4:** The apoptosis rate of liver tissue in each group of rats

Group	n	AI（%）
Sham	10	3.89±0.60
HIRI	10	27.66±4.76^*^
GL100 mg/kg	10	20.57±4.09^*#^
GL200 mg/kg	10	15.43±3.66^*#^

Compared with Sham group, ^*^P<0.05; compared with HIRI group, ^#^*P<*0.05

HIRI: Hepatic ischemia-reperfusion injury; GL: Glycyrrhizin

## Conclusion

GL has an obvious protective effect on HIRI, and its mechanism may be related to activation of the Nrf2/HO-1 signaling pathway, inhibition of oxidative stress, inflammation, autophagy, and apoptosis.
